# Interactions of melatonin with various signaling pathways: implications for cancer therapy

**DOI:** 10.1186/s12935-022-02825-2

**Published:** 2022-12-29

**Authors:** Ainaz Mihanfar, Bahman Yousefi, Bita Azizzadeh, Maryam Majidinia

**Affiliations:** 1 Solid Tumor Research Center, Cellular and Molecular Medicine Institute, Urmia University of Medical Sciences, Urmia, Iran; 2 Immunology Research Center, Tabriz University of Medical Sciences, Tabriz, Iran; 3 Department of Biochemistry, School of Medicine, Ilam University of Medical Sciences, Ilam, Iran

**Keywords:** Melatonin, Anti-cancer, Signaling pathways

## Abstract

Melatonin is a neuro-hormone with conserved roles in evolution. Initially synthetized as an antioxidant molecule, it has gained prominence as a key molecule in the regulation of the circadian rhythm. Melatonin exerts its effect by binding to cytoplasmic and intra-nuclear receptors, and is able to regulate the expression of key mediators of different signaling pathways. This ability has led scholars to investigate the role of melatonin in reversing the process of carcinogenesis, a process in which many signaling pathways are involved, and regulating these pathways may be of clinical significance. In this review, the role of melatonin in regulating multiple signaling pathways with important roles in cancer progression is discussed, and evidence regarding the beneficence of targeting malignancies with this approach is presented.

## Introduction

N-acetyl-5-methoxy tryptamine or melatonin is a neuro-hormone which is synthesized from the metabolism of L-tryptophan [1]. It is thought that this molecule was initially synthetized by primitive uni-cellular organisms in order to fade of the toxic effects of oxidant molecules in the environment, but has gained more sophisticated roles during evolution, such as regulating the day and night cycle, sexual selection, environmental tolerance and immunomodulatory roles [2, 3]. One important function of melatonin which is obtained early in the process of evolution and has been conserved during it is the ability of melatonin to affect various signaling pathways. This is done by both receptors mediated and non-receptor mediated pathways. Melatonin is able to affect the singling output of pathways involved in inflammation by acting as an anti-oxidant molecule. It can also activate its receptors located at the cellular membrane and in the nucleus of the cells, altering normal cellular functions and affecting the expression of key mediators of different signaling pathways [4].

**Fig. 1:**
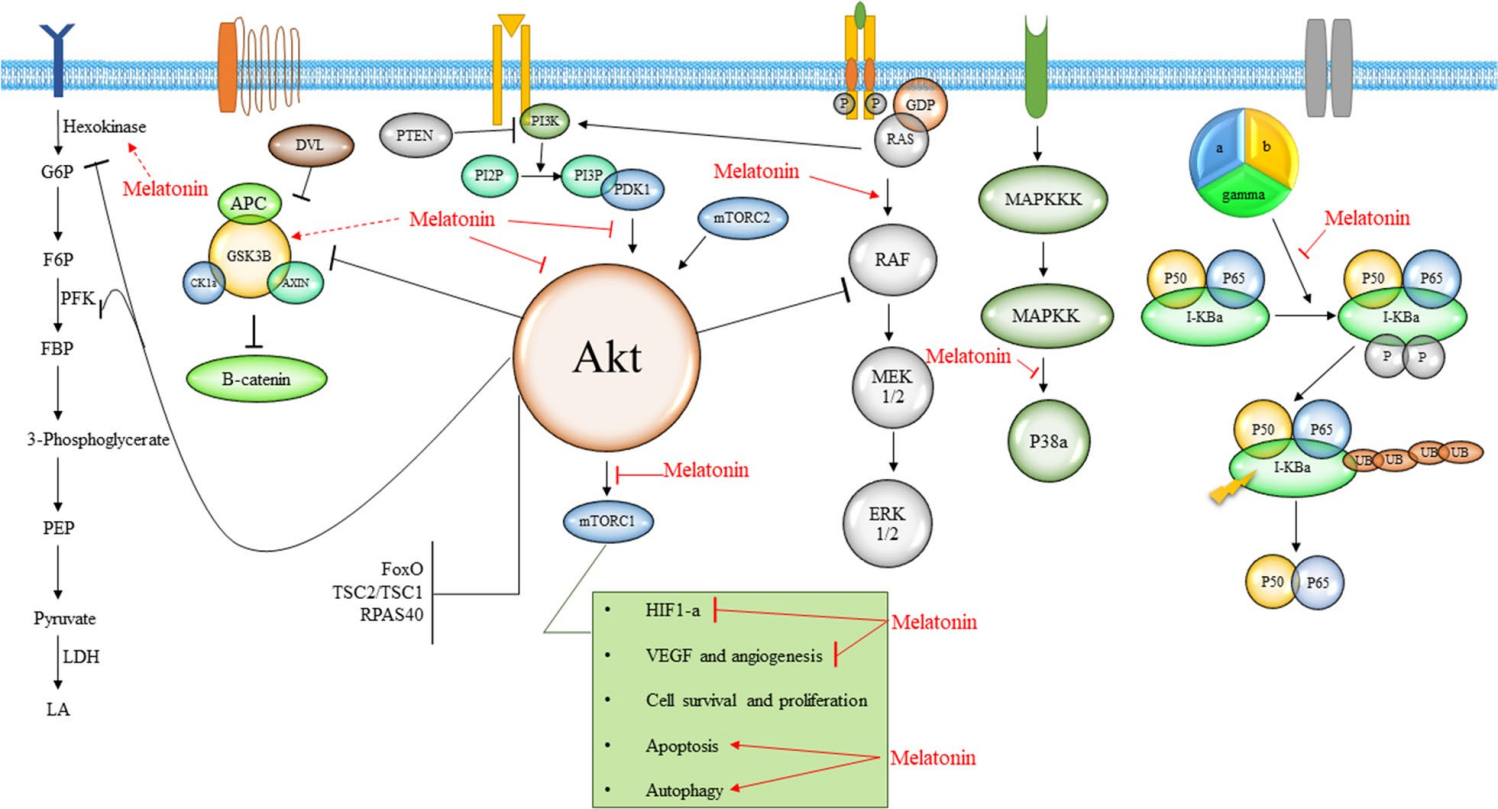
A summary of interaction between melatonin and various signaling pathways

As mentioned, there is a decisive relation between outcomes of melatonin administration and its effects on signaling pathways. This relation is further emphasized in pathologies in which these pathways are involved and are even considered as etiologic factors for disease emergence. One such condition is cancer [5, 6]. Cancer is characterized by uncontrolled cellular proliferation, evasion of apoptosis, cellular migration and metastasis and changes to normal intrinsic cellular functions, such as energy metabolism [7]. These characteristics are dependent on the abnormally increased or inhibited signaling outputs of pathways which are effective in the normal regulation of cell functions. Examples of these pathways are the PI3K/AKT/m-TOR pathway, growth factor signaling pathway, NOTCH pathway and more (Table 1). These interactions between malignant transformation and signaling pathways and the effects melatonin exerts on them, has led researchers to examine if melatonin has any significant anti-cancer effects mediated by regulating signaling pathways (Fig. 1).

**Table 1:** A comprehensive list of signaling pathways involved in the anticancer effects of melatonin and their molecular mechanism of action

Signaling pathway effected	Cell line	Melatonin dose/concentration	Major effect on carcinogenesis	Other findings	Refs.
IGF signaling pathway	Prostate cancer (PCa) cell line (LNCaP)	–	Reduced cancer cell proliferation	Upregulation of IGFBP3 and downregulation of IGF1R	[142]
VEGF signaling pathway	MDA-MB-231 and MCF-7 cell lines	1 mM and 1 ng/mL	Suppression of cancer cell growth and viability	Decrease in VEGF-A protein expression and increase in IGFBP-3, IGFPB-6, IGF-1, IGF-1R proteins	[143]
	MDA-MB-468 cells	0.01 mM, 0.1 mM and 1 mM	Suppression of angiogenetic features of breast cancer cells	Inhibition of expression of IGF-IR, HIF-1α and VEGF proteins through regulation of miRNA-152-3p	[144]
Notch signaling pathway	Glioblastoma cell lines (U251 and T98G)	100 µM and 1 mM	Inhibition of viability and self-renewal of glioblastoma stem-like cells (GSCs)	EZH2-Notch1 signaling pathway suppression	[98]
	MCF-7 breast cancer cells	100 µM	Inhibition of growth and viability of breast cancer cells	Altered conductance through Ca^2+^ and voltage-activated K^+^ (BK) channels and disruption of Notch1 signaling pathway	[99]
	Endometriotic eutopic epithelial cells (EEC) from patients’ tissues	1 mM	Suppression of 17β-estradiol-induced invasion, migration and epithelial to mesenchymal transition (EMT) of endometriotic cells	Decrease in the activity of the Notch signaling	[100]
	GC-1 spg cell line	0.1 µM	Growth inhibition of testicular germ cell tumor	Suppression of tumor growth through regulation of miRNA	[101]
NF-κB signaling pathway	HUVEC	10 µM	Inhibition of angiogenesis	Decrease in the production of MMP9 via inhibition of NF-κB signaling	[116]
	HepG2 cells	1 mM	–	Upregulation of NF-κB pathway proteins	[117]
	MDA-MB-231 cell line xenograft in Balb/c nude athymic mice	40 mg/Kg	Decrease in tumor size and growth	Decrease in expression of NF-κB pathway	[117]
	Human vulvar‐derived steroid independent leiomyosarcoma SK‐LMS‐1 cells xenograft in athymic, inbred nude rats	75 μg	Suppression of aerobic glycolysis (Warburg effect), survival and tumor growth	Suppression of activation of ERK1/2, AKT, GSK3β and NF-kB (p65)	[31]
Warburg Effect	Ewing sarcoma CL: A-673, TC-71 and A-4573	0,1 mM incubation for 2,4,6,8 h	Reversal in metabolic profile	Increased glucose uptake, LDH activity, lactate production and HIF-1α activation / Not effective on chondrosarcoma cells	[175]
Warburg Effect, Suppressed phospho-activation of ERK 1/2, AKT, GSK3β and NF-kB (p65)	Leiomyosarcoma CL: SK-LMS-1	100 nM–1 pM incubation for 6 days	Repressed cell proliferation and cell invasion	Suppressed aerobic glycolysis, complete inhibition of tumor linoleic acid uptake, 13-HODE release, as well as significant reductions in tumor cAMP levels, DNA content and [(3) H]-thymidine incorporation into DNA	[31]
mTORC1/ribosomal protein S6 kinase beta-1 (p70S6K)/ribosomal protein S6 (RP-S6) pathway	Hepatocellular carcinoma CL: Hep3B	2 mM	Preventing HIF-1α synthesis to block the cytoprotective mitophagy induced by the hypoxic microenvironment, reduced resistance to sorafenib	Enhanced Akt phosphorylation by the mTORC1/C2 negative feedback	[34]
mTOR/Akt	Hepatoma H22	10 & 20 mg/kg	Melatonin triggers an autophagic process by enhancing Beclin 1 expression and inducing a conversion of microtubule-associated protein 1 light chain 3(LC3)-I to LC3-II	The autophagy inhibitor, 3-methyladenine(3-MA), significantly enhanced the melatonin-induced apoptosis in mouse hepatoma H22 cells	[35]
mTOR/Akt	Head and neck SCC CL: Cal-27 and SCC-9	0.1, 0.5 or 1 mmol/L melatonin combined with 20 nM rapamycin	Decreased cell viability, proliferation and clonogenic capacity, increased ROS production, increasing apoptosis and mitophagy	Bined treatment with rapamycin and melatonin blocked the negative feedback loop from the specific downstream effector of mTOR activation S6K1 to Akt signalling	[37]
ERK1/2 and p38 MAP kinases	Hepatoma cells H4IIE	0–5.0 mM	Inhibit the effects of H2O2-induced oxidative stress	Attenuated H_2_O_2_‐induced activation of the ERK1/2 and p38 MAP kinases	[45]
PI3K/Akt/mTOR pathway	Melanoma cell B16F10	0–1.0 mM	Reduced cell viability	Cell viability was significantly decreased after treatment with melatonin combined with ER stress from thapsigargin or tunicamycin compared to no treatment or treatment with melatonin only	[46]
RTK/PKC/Akt/NF-kappaB pathway	C6 glioma cells	Intraperitoneal administration of 15 mg/kg	Inhibition of cell growth	Increase of basal redox state	[48]
p38 MAPK	Breast cancer CL: MCF-7/6, MCF-7/Her2.1, and MCF-7/CXCR4 cells	1 nM	Suppression of the invasive potential	Repressed the proteinase activity of MMP-2 and MMP-9	[62]
Akt/ GSK3β	Breast cancer MCF-7, MCF-7/ steroid receptor negative	Circadian cycle of plasma melatonin level was assessed	Suppression of EMT		[63]
MAPK/JNK	Prostate cancer LNCaP	0–3 mM incubation for 0–48 h	Apoptosis	Melatonin-induced apoptosis was JNK- and p38-dependent, but ERK-independent	[65]
MAPKs/ERK/JNK	HepG2 human hepatocarcinoma cells	1 and 2.5 mM	Cell viability	Both melatonin concentrations increased the expression of phosphorylated p38, ERK, and JNK. ERK activation was completely abolished in the presence of luzindole	[66]
JNK/MAPK	Lung cancer A549	0.1–5 mM incubation for 24 h	Cell migration was reduced to about 20%	The expression level of OPN, MLCK and phosphorylation of MLC of A549 cells were reduced, while the expression of occludin was conversely elevated, and occludin located on the cell surface was obviously increased. The phosphorylation status of JNK in A549 cells was also reduced when cells were treated by melatonin	[67]
P38 MAPK	Human melanoma SK-MEL-1 cells	0–1 mM incubation for 24–72 h	Decreases cell proliferation and induces melanogenesis	Comparative studies with known antioxidants such as N-acetyl-l-cysteine and trolox indicate that the growth of SK-MEL-1 cells is highly sensitive to antioxidants	[68]
P38 MAPK/JNK/ERK	Gastric cancer cell line (AGS)	0,1,2 mM incubation for 0–72 h	Apoptosis, enhancing the anti-tumour effects of cisplatin, with low systemic toxicity	Increased caspase-3 cleavage and Bax protein expression and decreased Bcl-2 protein expression in a time-dependent manner	[69]
P38 MAPK/Akt/ERK/JNK	RCC cells (Caki-1 and Achn)	0.5–2 mm	Reducing metastasis potential	Inhibition of MMP-9	[176]
JNK/SP-1 signaling	Nasopharyngeal carcinoma	0,0.5,1.0 mM	Suppression of the motility of NPC	Regulating TPA-induced MMP-9 gene expression via inhibiting SP-1-DNA binding ability, the c-Jun N-terminal kinase/mitogen-activated protein kinase pathway is involved in the melatonin-mediated tumor suppressor activity	[70]
Rho-associated protein kinase (ROCK)	MCF-7 cells	1 nm	Reduces migration and invasiveness	Increased expression of two cell surface adhesion proteins, E-cadherin and beta(1)-integrin, inhibitory effects on cell migration by changing cytoskeletal organization of leader MCF-7 cells	[72]
Wnt pathway and Raf/MEK/ERK pathway	UC3 bladder cancer cells	Cotreatment with melatonin 1 µM and VPA 5 mM incubation for 24 h	Apoptosis, autophagy and necrosis	Increased the expression of endoplasmic reticulum (ER)-stress-related genes, enhanced the expression of E-cadherin, and decreased the expression of *N*-cadherin, Fibronectin, Snail and Slug	[84]
miRNAs	MCF-7 human breast cancer cells	1 and 100 nM	22 miRNAs were differentially expressed in melatonin-treated MCF-7 cells depending on the concentration of melatonin treated with	–	[158]
miRNAs	PC-3 prostate cancer cells	1 mM incubation for 4 h under hypoxia	33 miRNAs (> 2 folds) including miRNA3195 and miRNA 374b were significantly upregulated and 16 miRNAs were downregulated in melatonin-treated PC-3 cells under hypoxia compared to untreated control upregulation of miRNA3195 and miRNA374b mediates anti-angiogenic property	Melatonin significantly attenuated the expression of hypoxia-inducible factor (HIF)-1 alpha, HIF-2 alpha and vascular endothelial growth factor (VEGF) at mRNA level in hypoxic PC-3 cells melatonin enhanced the expression of miRNA3195 and miRNA 374b in hypoxic PC-3 cells	[159]
miRNAs	HCT 116 and MCF-7 cells	Incubation for 0–72 h, concentration not mentioned clearly	Decrease in miR-24 levels post-transcriptionally affecting cell proliferation, DNA damage, RNA metabolism and cell shape and transformation	miR-24 is upregulated in colon, breast and head and neck datasets and its levels negatively correlate with overall survival	[160]
miRNAs	Human glioma cell lines U87, U373 and U251	100 μM, 1 μM and 1 nM	Promoting cell apoptosis and repressing cell proliferation, migration and invasion	Downregulating the expression of miR-155 via repression of c-MYB	[161]
miRNA	Gastric cancer	0,1,5 µM incubation for 0–72 h	Apoptosis, enhancing the expression of miR-16-5p	miR-16-5p targeted Smad3 and consequently negatively regulated the abundance of Smad3	[162]

## Anti-cancer effects of melatonin

Much consideration has been given towards the anti-cancer effects of melatonin. Early studies had found that melatonin could inhibit cell proliferation in in vitro cultures of malignant cells, but the exact mechanism of this effect was not known [4]. More studies clarified that melatonin exerts its anti-cancer effects in both direct and in-direct methods. Indirectly, it acts as a free radical scavenger and can be used as a chemopreventive agent for cancer [8]. It can further protect the myeloid system of the bone marrow, and help regulate the immune system, contributing to an optimal immune response to the tumor [9]. Melatonin is shown to potentiate cellular immunity by increasing the secretion of interleukin-2, interleukin-10 and interferon-γ, which in term activate the T cells [10]. Melatonin also contributes to tumor behavior by interacting with the tumor microenvironment, which has critical functions in suppressing or promoting carcinogenesis [11]. A study by Sonehara et al. showed that melatonin was able to promote apoptosis in breast cancer cells being under constant acidosis [12].

The direct anti-cancer effects of melatonin consist of its effects on cell functions such as cellular proliferation and apoptosis, and processes such as angiogenesis. Melatonin is shown to affect DNA damage response (DDR), a signaling pathway which can ultimately control cell proliferation and apoptosis [13]. It is shown that aberrations in DDR are associated with cancer, and melatonin is a beneficial agent to reverse the effects of these aberrations [14]. Other studies also showed that melatonin could interact with the direct mediators of cell cycle arrest, apoptosis and autophagy, preventing the survival of neoplastic cells.

Melatonin is also effective in decreasing the angiogenesis which is initiated by the malignant cells. As tumors enlarge, they outgrow their pre-existing vasculature, and end up in an environment with low concentrations of oxygen and increased concentrations of cellular waste and debris. These changes stimulate the malignant cells to initiate angiogenesis, which is mediated by the activation of Hypoxia-inducible factor 1 and the subsequent increase in the amounts of vascular endothelial growth factor (VEGF) [15]. These two mediators initiate a process which leads to formation of new vessels with leaky membranes, which enable uncontrolled passage of substances and cells, ultimately facilitating distant metastasis, and also enable malignant cells to overcome the harsh environment [16]. Melatonin is shown to be a potent inhibitor of angiogenesis, by decreasing the expression of VEGF, HIF-1 and the regulation of other mediators [17].

Another mechanism by which melatonin inhibits cancer progression is by antagonizing the effect of sexual hormones on hormonal receptors, such as those found on breast cancer cells, ovarian cancer cells and prostate cancer cells [18]. Stimulation by sex hormones has been proven to increase the cellular proliferation of the aforementioned cancers, and some therapy regimens consist of more conventional agents with anti-estrogenic effects, such as tamoxifen [19]. Recent proposed therapy regimens have mixed melatonin with tamoxifen and similar agents, with the results showing a promise for more comprehensive trials in the future [20].

## PI3K/Akt/mTOR signaling pathway

PI3P is the phosphorylated form of phosphatidylinositol 4,5 diphosphate (PI2P) by PI3K and is dephosphorylated by PTEN, a tumor suppressor protein with the major inhibitory effects on the pathway [21]. PDK1 activated by the PI3P, phosphorylates Akt, which is also activated by mTOR complex 2. Akt in turn activates mTOR complex 1 in two steps by inactivating both TSC2 and PRAS40. At last, mTOR complex 1 induces divergent cascades leading to different outcomes [22].

### Targeting in cancer

The PI3K/Akt/mTOR pathway has a crucial role in cell survival and proliferation as well as in, differentiation, apoptosis, tumorigenesis, angiogenesis, autophagy and metastasis by means of attenuating epithelial-mesenchymal transition (EMT). Ginkgolic acid and curcumin have been used to inhibit the PI3K/Akt/mTOR axis of EMT in lung cancer metastasis [23, 24]. Inhibition of the PI3k/Akt/mTOR pathway by aflatoxin B2 and *E. adenophorum* leads to pro-autophagic state and apoptosis in the hepatocytes [25]. An in vitro study revealed that by inhibiting the pathway, apigenin induces apoptosis in the hepatocellular carcinoma cells [26]. Reviews have proposed that inhibitors of the pathway may be efficacious in prevention or treatment of age-related macular degeneration or proliferative diabetic retinopathy [27, 28]. The blockade of PI3K by LY294002 results in G1 cell cycle arrest having synergistic effect with chemotherapeutics acting in this phase and antagonistic encounter with therapeutics interfering S or G2 phase [29, 30].

### Interaction with melatonin

Melatonin possesses multiple roles within the intracellular signaling pathways. It can inhibit the Warburg effect in leiomyosarcoma and Ewing sarcoma cells [31]. Selective mTOR inhibitors such as everolimus are approved for treatment of renal cell carcinoma, subependymal giant cell astrocytoma, progressive neuroendocrine tumors of the pancreas and hormone receptor positive breast cancer. Furthermore, inhibition of mTOR causes hyper-activation of MAPK pathway through feedback loops suggesting that combined use of inhibitors of the two pathways may be more efficient in cancer treatment [32]. Presence of these feedback loops makes it difficult to predict the outcomes of suppression of these pathways [33].

mTOR and Akt phosphorylation and the production of hypoxia induced factor-1α are inhibited by melatonin [34–36]. PI3K/Akt/mTOR pathway can be suppressed by combination of rapamycin and melatonin, which was used in vitro to inhibit head and neck squamous cell carcinoma proliferation with satisfactory results; melatonin reduced the toxicity of rapamycin on the normal cells and enhanced the antitumor activity of rapamycin [37]. A very important mechanism of melatonin induced autophagy and apoptosis is the modulation of the cellular response to oxidative stress-induced endoplasmic reticulum (ER) stress [38]. Cellular stress causes accumulation of misfolded proteins by means of ER stress, which leads to a series of reactions known as unfolded protein response (UPR). In short-term UPR protects the cell by regaining the intracellular homeostasis [39]. However, in the long-term it can lead to several types of diseases like diabetes, neurodegeneration and cancer [40]. Melatonin seems to regulate the UPR in a contradictory manner, having pro-apoptotic function in the tumor cells and anti-apoptotic function in the normal viable cells [41]. In general melatonin is known to prevent apoptosis in neurodegenerative and immunologic diseases and increase it in cancerous cells [42–44]. Melatonin is claimed to be involved in the autophagy and apoptosis of several types of cancer cells such as hepatocellular carcinoma, melanoma and ovarian carcinoma via PI3K/Akt/mTOR pathway and ER stress response [35, 45–47]. Treating glioma cells with melatonin causes cycle arrest in G1 to S phase by inhibiting Akt and NF-κB but not ERK [48]. In several cancers, adding melatonin causes reduction in resistance to chemotherapy regimen and its systemic toxicity [49]. However, it appears that melatonin is not as safe as it was thought to be as it can lead to ROS mediated mitochondrial damage-induced apoptosis in the platelets which may lead to elevated risk of thrombosis in long term use of high dose melatonin [50].

## MAPK signaling pathway

The pathway has been conserved through evolution and has several key interface members such as the RAS superfamily, RAFs, ERK, MEK 1/2, JNKs [51, 52]. MAPK itself translocates into the nucleus to continue the cascade [53, 54]. Activation of the downstream effectors leads to nuclear responses mediated by transcription factors like cMyc. Inhibition of ERK/MAPK and PI3K/Akt/mTOR signaling pathways leads to activation of FOXO, a nuclear transcription factor and apoptosis in the pancreas cancer cells [55]. The nuclear response may lead to cell proliferation, migration, apoptosis, differentiation, angiogenesis, metastasis [56].

### Targeting in cancer

The ERK/MAPK pathway is one of the most important regulators of cell proliferation. Mutations in B-RAF and RAS tend to be oncogene, while stress-activated JNK and p38 mostly confront neoplastic transformations [57]. Dysregulation in the MAPK pathway is implicated in the evolution of melanoma, colorectal cancer, hepatocellular carcinoma. RASs can activate the PI3K/Akt/mTOR pathway by phosphorylating PI3K [33]. Broad substrate specificity of Akt and ERK enzymes gives place to crosstalk between the two signaling pathways [58]. These crosstalks form a big network of feedback loops that should be carefully considered when manipulating one member of the pathways which may cause unwanted negative effects. Exosomes released by gastric tumor cells and bone marrow mesenchymal stem cells contain wide spectrum of proteins and mediators that can in part, stimulate proliferation of the adjacent tumor cells via PI3K/Akt/mTOR and ERK/MAPK and seem to be an important mechanism for intercellular communication [59]. An important association of the MAPK signaling pathway is the Rho-dependent protein kinase (ROCK) signaling pathway which alters the cytoskeleton microtubules and microfilaments regulating cell shape and migration [60].

### Interaction with melatonin

By inhibition of p38 MAPK, melatonin suppresses EMT and metastasis of breast cancer cells [61]. It also inhibits cellular proliferation and invasive potential of estrogen receptor α positive cells by repressing phosphorylation of p38 MAPK [62]. Glycogen synthase kinase 3β, an important mediator in cell survival and proliferation, has a circadian rhythm of phosphorylation in breast cancer cells. Melatonin activates this enzyme by inhibiting Akt phosphorylation, inducing β-catenin degradation and inhibiting EMT. Thus, light-at-night may lead to advanced stages of cancer by reducing melatonin production from the pineal gland during night [63]. Among breast cancer patients, disruption in the circadian production of melatonin is related to intrinsic resistance to tamoxifen; thus, supplementary melatonin may be of therapeutic value [64]. By activation of p38 kinase and JNK, melatonin induces apoptosis in the prostate cancer cells in a dose-dependent manner, while ERK is not responsive to treatment with melatonin [65]. In vivo treatment of hepatocellular carcinoma cells with high concentrations of melatonin results in cell cycle arrest and apoptosis via MAPK signaling pathway [66]. Lung adenocarcinoma cells showed up-regulated expression of occludin and reduced phosphorylation of JNK leading to significantly reduced migration potential of the cells within 24 h of treatment with melatonin [67]. Melatonin reduces cell proliferation in melanoma by activation of p38 kinase [68]. Ovarian cancer cells showed attenuation in expression of p38 kinase, Akt and mTOR following treatment with melatonin [47]. Through a similar mechanism, melatonin induces apoptosis in gastric cancer cells [69]. By suppressing Akt/MAPK signaling pathways and inhibiting matrix-metalloproteinase (MMP)-9 transactivation, melatonin reduced EMT and metastasis in renal cell carcinoma [70]. Melatonin reduced MMP-9 expression in the nasopharyngeal carcinoma and oral carcinoma by inhibiting binding affinity of transcription factor SP-1 to DNA; this led to reduced cell migration potential and EMT [70, 71]. Melatonin also inhibits the ROCK signaling pathway and consequently cell migration and metastasis ability of tumor cells [72]. Finally, JNK is altered through crosstalk between the MAPK and the Wnt pathways [73].

## Wnt/β-catenin signaling pathway

The signaling pathway has been conserved through evolution because of its crucial roles in many cellular functions, including but not limited to embryonal anterior–posterior axis development. Three main branches of the pathway have been introduced, in most of which, β-catenin is considered to be the central mediator. These pathways require two additional co-receptors along with Wnt, Frizzled proteins and LRP 5 or 6 [74].

### Targeting in cancer

Activation of the pathway in turn, activates the downstream disheveled protein, which inhibits the β-catenin-destruction complex including APC protein, the initiator of colon cancer evolution. In the absence of Wnt ligand, β-catenin undergoes degradation by ubiquitin–proteasome complex [75]. β-catenin translocates into the nucleus and after binding with other transcription factors, activates expression of important proteins (canonical pathway). A very important second messenger of the pathway independent of β-catenin is calcium which eventually activates calcium/calmodulin-dependent kinase II, protein kinase C, phospholipase C and phosphodiesterase that rises a converge of responses (Wnt/calcium pathway) [76]. Within the tumor bulk there is heterogeneity regarding the activity of the Wnt signaling pathway with predominant activity in the active margin boosting the invasion machinery of the tumor [77]. Wnt signaling pathway is upregulated by autocrine mechanisms in the tumor cells. Dysregulation in circadian rhythm of light and dark is a known factor in development of cancers. In vivo studies are underway to target the Wnt pathway with anti-cancer purposes. Two small biologic molecules have shown capability of reversible inhibition of the pathway [78]. Resveratrol, a polyphenol inhibits cell proliferation and induces autophagy in breast cancer stem cells via suppressing the Wnt/β-catenin signaling pathway [79]. A study revealed that Wnt pathway dysregulation has a part in EMT in the malignant breast cancer cells and inhibition of the pathway by sFRP-1 caused decreased cell proliferation, motility and reduced lung metastases in mice models [80]. Monoclonal antibodies, anti-cancer viral therapeutics and agonist/antagonist peptides are under development to target the members of the pathway [81–83]. Interestingly, aspirin reduces colorectal cancer cell growth partially by inhibiting Wnt/β-catenin pathway [83].

### Interaction with melatonin

Melatonin in combination with valproate acid showed synergistic effect with bladder cancer chemotherapy. It was revealed that these two add-ups induced expression of genes related to ER stress, autophagy, apoptosis and necrosis via activation of Wnt and MEK/ERK pathway and upregulation in the E-cadherin which may have a role in reducing the metastasis virulence of the tumor cells and suppression of EMT [84]. Accordingly, a switch in the expression of E-cadherin to N-cadherin has a pivotal role in EMT and progression of prostate cancer [85]. In nasopharyngeal carcinoma, melatonin not only reversed cisplatin chemoresistance, but also enhanced cisplatin antitumor activity by suppressing the nuclear translocation of β-catenin, and reducing expression of Wnt/β-catenin response genes [86]. In addition, melatonin suppresses chronic restraint stress-mediated metastasis of epithelial ovarian cancer via NE/AKT/β-catenin/SLUG axis [87]. Li et al. reported that melatonin inhibited the biological functions of osteosarcoma cells by repressing the expression of lncRNA JPX through regulating the Wnt/β-catenin signaling pathway, which indicated that melatonin might be applied as a potentially useful and effective natural agent in the treatment of osteosarcoma [88].

## Notch signaling pathway

Notch signaling pathway has a key role in regulation of cell-to-cell communications during important mechanisms such as embryogenesis, cellular proliferation, differentiation, and apoptosis. This signaling pathway is also critical for proper hematopoiesis, regulation of the immune system, breast development, colorectal epithelial maturation and neural stem cell survival. Dysregulation of the Notch signaling pathway is involved in pathogenesis of many cancers [89].

### Targeting in cancer

It has been noted that Notch signaling pathway has crosstalk with growth factors in breast cancer, androgen-dependent signals in prostate cancer and Hedgehog pathway in pancreatic cancer [90, 91]. Regarding the role of this pathway in some cancers, targeting of this pathway has been considered in treatment of cancers. Some types of drugs have been tested in clinical trials for some selected cancers like T-cell acute lymphoblastic leukemia, in which mutations related to Notch signaling are involved in pathology of cancer [92]. The primary action for development of target therapy of Notch in cancer, is inhibition of γ-secretase mediated Notch cleavage. Until now, γ-secretase inhibitors (GSIs) have been designed for treatment of T-cell acute lymphoblastic leukemia and estrogen receptor-positive breast cancer [93]. In a study by Rizzo et al.; it is demonstrated that estrogen can inhibit Notch activity through inhibition of γ-secretase activity, thus estrogen blockade by antiestrogens or aromatase inhibitors could lead to success in treating breast cancer with GSIs [94]. Furthermore, after increase in the knowledge about Cancer Stem Cells (CSC), it is noted that single-agent GSI therapy may be a useful treatment in triple-negative breast cancer because of its CSC-like characteristics [95]. Moreover, DLL4 monoclonal antibodies are designed against cancers through disruption in their angiogenesis, because DLL4 is a Notch ligand involved in the process of angiogenesis [96]. Other agents for Notch-targeted therapy of cancer are MAML inhibitors which can target mastermind-like (MAML)-CSL-Notch complex formation. In addition to the above, selective inhibition of Notch receptors may reduce intestinal toxic effects and off-target adverse effects like diarrhea resulting from Notch inhibition [97].

### Interaction with melatonin

In a study by Zheng et al.; it is shown that melatonin can play a role against glioblastoma through inhibiting the viability and growth of Glioblastoma stem-like cells (GSCs). This event occurs via EZH2-Notch1 signaling pathway suppression, which is mediated by melatonin [98]. In another study by Margheri et al.; it has been seen that melatonin in combination with all-trans retinoic acid and somatostatin can inhibit growth of MCF-7 breast cancer cells via disruption of Notch1 signaling pathway. In this study they noted that the levels of Notch1 were significantly downregulated in the melatonin-treated cells [99]. Furthermore, in a study it is revealed that melatonin, a therapeutic agent for endometriosis, can suppress 17β-estradiol-induced invasion, migration and epithelial to mesenchymal transition (EMT) in endometriotic cells of endometrium, because of melatonin-mediated decrease in the activity of the Notch signaling pathway [100]. Also in a study related to melatonin effects on miRNA expression profiles in GC-1 spg cell line, pathway analysis indicated that these effects of melatonin on miRNAs involved in cancers and signaling pathways, such as Notch and others. This indicated that melatonin can even play a therapeutic role in testicular germ cell tumors by interfering with Notch signaling pathway [101].

## NF-κB signaling pathway

The nuclear factor κB (NF-κB) concluding a family of transcription factors (RelA (p65), RelB and c-Rel, and the precursor proteins NF-κB1 (p105) and NF-κB2 (p100)) which are bind to κB sites in promoters and enhancers of a variety of genes and induce or repress their transcription [102]. The NF-κB signaling pathway is a critical pathway for regulation of immunity and other vital mechanisms of the cell like differentiation, proliferation and survival. Hence, its dysregulation results in inflammatory disorders, autoimmune and metabolic diseases and cancer development [103].

### Targeting in cancer

It is shown that NF-κB signaling pathway is activated in many cancers such as breast cancer and it is related to some oncogenic characteristics of tumor cells and their resistance to chemotherapy or radiation [104, 105]. It is perceived that NF-κB activity is maintained in cervical cancer cells through activation by Notch signaling [106]. Also, in gastric cancer, Rel-A (p65), a member of NF-κB signaling pathway transcription factors, correlated with tumor invasion-related features like lymphatic invasion of tumor cells, depth of invasion, tumor size and peritoneal metastases [107]. Considering the mentioned cases, utilization of therapeutic potential of this signaling pathway is implicated. It is noted that inhibition of NF-κB pathway improves the apoptotic response to treatments such as radiation therapy or chemotherapeutic drugs like Taxol [108]. Another approach is designation of Proteasome inhibitors for targeting NF-κB signaling pathway, because they cause blockade in the degradation of proteins which are necessary for activation of NF-κB signaling such as IκBs, NF-κB1/p105 or NF-κB2/p100 and by this way they can prevent NF-κB activation [109]. Also in a study by Vequaud et al.; it has been reported that Survivin, a target gene for cancer therapy, plays its therapeutic role in breast cancer through its modulating effect on NF-κB signaling pathway and autophagy [110]. Inhibitors of apoptotic proteins (IAPs) are overexpressed in some cancers. IAPs have differential effects on the NF-kB pathway. They can be good options for targeting NF-kB in cancer. Synthetic peptide-mimetic compounds (IAP antagonists or SMAC mimetics) which can bind to IAPs, prevent activation of NF-kB signaling. Thus, SMAC-mimetics represent a targeted therapeutic approach for cancer therapy. The SMAC mimetic Birinapant, showed anti-cancer activity in both hematologic malignancies and solid tumors [111, 112]. Furthermore, Curcumin is a natural compound that inhibits the kinase activity of IKKb in the NF-κB signaling pathway. It is used in treatment of mantle cell lymphoma, colon cancer and advanced pancreatic cancer [113, 114].

### Interaction with melatonin

It has been noted that melatonin inhibits the activation of NF-κB and this is one of the antioxidant effects of melatonin, because some second messengers of NF-κB signaling are known as free radicals [115]. Melatonin can reduce the production of MMP9 via inhibition of NF-κB signaling; this can affect the cell migration and invasion of glioma cells and other tumors [116]. In a study it has been shown that melatonin could upregulate the NF-κB pathway proteins expression in hepatocarcinoma cells, but treatment of breast cancer cells with melatonin causes decrease in expression of NF-κB pathway proteins in them [117]. In another study by Mao et al.; it has been demonstrated that melatonin can suppress the aerobic glycolysis (Warburg effect), survival and tumor growth in leiomyosarcoma cells through modulating some signaling pathways such as NF-κB pathway [31]. On the other hand, there is a concept known as immune-pineal axis. In this concept, NF-κB can inhibit melatonin synthesis in pinealocytes and induces melatonin synthesis in macrophages, but melatonin reduces NF-κB activation in pinealocytes and immune competent cells. This balance could be affected in the pathological conditions that disrupt melatonin rhythms [118].

## JAK/STAT signaling pathway

The Janus kinase/signal transducers and activators of transcription (JAK/STAT) pathway is a vital signaling mechanism, including pleiotropic cascades which are important for secretion of a wide array of cytokines and growth factors. The STAT proteins are a family of transcription factors in the cytoplasm, activated by JAKs via phosphorylation of tyrosine residues [119]. The cascades of this pathway are necessary for hematopoiesis, development of the immune system, adipogenesis, mammary gland development and lactation and many other processes. Occurrence of these events depends on JAK activation, because of its important role in stimulation of cell proliferation, differentiation, cell migration and apoptosis [120]. Mutations that cause dysregulation of JAK signaling can result in many diseases such as leukemia and other cancers.

### Targeting in cancer

The inappropriate activation of the JAK/STAT signaling pathway can directly result in oncogenesis [119]. The cancers in which the aberrant activation of this signaling pathway was seen, including breast, prostate, pancreatic, colorectal and other cancers. Because of the importance of this pathway, it becomes a therapeutic target for cancer treatment. It is shown that anti-cancer effects of (−)-Epigallocatechin gallate (EGCG), the most abundant catechin in tea, are performed through inhibition of phosphorylation and expression of both JAK3 and STAT3 proteins [121]. Also EGCG treatment for cancers such as breast cancer, has been shown that inhibit the activity of STAT3 [122]. Another therapeutic agent which plays its role against cancer cells through inhibition of JAK/STAT signaling pathway is Cucurbitacin I, which is a member of tetracyclic triterpenoids family known as cucurbitacins. This agent is used in treatment of anaplastic large cell lymphoma, lung carcinoma and glioblastoma multiforme via targeting of STAT3 from JAK/STAT pathway [123, 124]. Cucurbitacin B is another agent from this family which is used for treatment of leukemia and pancreatic cancer by the same mechanism as Cucurbitacin I [125].

### Interaction with melatonin

Maybe one of the important interactions of melatonin with the JAK/STAT signaling pathway is in the function of immune cells, especially macrophages. It was noted that melatonin affects the signaling pathways such as JAK/STAT and NF-κB pathways in macrophages. So it can be concluded that melatonin modulates the development of various macrophage-associated diseases, such as cancer [126]. Also it was seen that melatonin reduces the angiotensin II-related injury and apoptosis of podocytes in the diabetic nephropathy by inhibition of JAK/STAT signaling pathway [127]. In another study it was shown that melatonin can interfere with IGF-1 secretion levels of the liver by modulating the JAK2/STAT3 pathway [128]. Furthermore, it was reported that neuroprotective effects of melatonin in brain ischemia and reperfusion injury after it, are related to some signaling pathways including JAK/STAT signaling pathway [129]. On the other hand, it is demonstrated that JAK/STAT3 signaling pathway (after activating by leptin) can increase the expression of miR-7, which acts as a negative regulatory molecule inhibiting RAF1/MEK/ERK signaling pathway and results in decreased melatonin synthesis [130].

## IGF, VEGF, FGF, PDGF signaling pathway

The IGF (insulin-like growth factor) signaling pathway is a critical signaling pathway for somatic growth, acts through promoting cellular proliferation and differentiation. It also plays a powerful role in cell survival and prevention of apoptosis. These anti-apoptotic and pro-survival effects of this pathway cause its importance and vital role for cancer cell growth, development of cancers, and tumor resistance against some treatments [131]. Also VEGF family including vascular endothelial growth factors and their receptors, are key proteins for significant biological processes such as hematopoiesis, lymphangiogenesis and vascular permeability; Moreover, they can induce neovascularization and angiogenetic characteristics of tumors for example in breast cancer or colorectal carcinoma. Fibroblast growth factor (FGF) and platelet-derived growth factor (PDGF) are other growth factors playing a similar role in cell survival and growth. They are vital proteins for maintaining the cells healthy. On the other hand, FGF and PDGF have necessary roles for facilitation of tumor growth and metastasis via angiogenesis. Also they affect tumor survival by some interactions together [132].

### Targeting in cancer

Regarding to the above-mentioned proofs, it is perceived that signaling pathways of growth factors have important roles in development of many cancers. Hence, targeting these pathways can be a promising approach for treatment of cancer [133]. In this relation, therapeutic effects of targeted therapy for these pathways are shown in many clinical trials [134]. In the last decades of the twentieth century it has been shown that antibodies blocking the VEGF pathway can suppress tumor growth and angiogenesis. Bevacizumab, a monoclonal antibody against VEGF, is the first anti-angiogenic agent approved by the Food and Drug Administration (FDA). It is used in combination with 5-fluorouracil-based chemotherapy regimens for treatment of previously untreated metastatic colorectal cancer [135]. It is also used against non-small cell lung cancer and metastatic breast cancer. Other agents inhibiting the members of VEGF family and can be used in treatment of cancers, are including VEGF-Trap and VEGF-AS. Another approach for utilization of therapeutic potential of this pathway is inhibition of VEGFR kinase activity. Sunitinib is a small-molecule receptor tyrosine kinase inhibitor, which could inhibit PDGF receptor-β and acts as a FDA approved treatment for patients with gastrointestinal stromal tumors (GISTs). Other samples of these drugs are Sorafenib for metastatic renal cell carcinoma and Vatalanib for metastatic colorectal cancer [136, 137]. Also it has been demonstrated that neutralizing monoclonal antibodies against VEGFRs decreases primary and metastatic tumor growth via inhibiting VEGF signaling [138]. Furthermore, in a study by Lovly et al.; it is shown that combination therapy with Crizotinib, a selective tyrosine kinase inhibitor, and an IGF-1 receptor (IGF-1R)-inhibitor has therapeutic effect in lung cancer via sensitizing the cancer cells to Crizotinib by inhibiting the IGF signaling pathway activation [139]. Also there are other drugs such as Ganitumab and Dalotuzumab designed to treat cancer, using the mechanism of inhibition of the IGF pathway (especially by targeting the IGF-1 receptor) [140]. Moreover, in relation to FGF pathway inhibition for treatment of cancers, can refer to agents such as Dovitinib, Pazopanib, Ponatinib and other similar agents that are designed for treating breast, lung, ovarian and other cancers [134, 141].

### Interaction with melatonin

It is shown that melatonin can affect tumorigenesis of some cancers by modulating growth factors signaling pathways in the cancer cells. It is shown that anti-tumor activity of melatonin in prostate cancer is mediated by IGF pathway in such a way that IGFBP3 is upregulated but IGF1R is downregulated after treatment of cancer cells by melatonin [142]. In another study, it was seen that melatonin treatment of breast cancer cells causes reduced VEGF-A protein expression and increased IGFBP-3, IGFPB-6, IGF-1, IGF-1R proteins in those cells and results in suppression of cancer cell growth [143]. Other study related to crosstalk between melatonin and growth factors signaling pathway in cancer, is done by Marques et al.; in that study it is seen melatonin suppresses angiogenic features of triple negative breast cancer cells by inhibiting expression of IGF-IR, HIF-1α and VEGF proteins through regulation of miRNA-152-3p [144].

## Hedgehog pathway

The Hedgehog (Hh) pathway is one of other important pathways that controls vital mechanisms such as tissue polarity and stem-cell maintenance. In many studies it is demonstrated hyper-activation of this pathway causes tumorigenesis in a wide variety of tissues [145]. Also it is indicated that this pathway has relation with cancer stem cells in CML and breast cancer. It is shown inhibition of this pathway in pancreatic cancer can cause suppression of EMT and metastatic features of these cells [146]. One of the inhibitors of this pathway is Cyclopamine, a plant-derived steroidal alkaloid, which is an antagonist for Smo in the Hh pathway. Furthermore, Robotnikinin is another inhibitor of this pathway that can act as an inhibitor of extracellular sHh; it is also one of small synthetic molecules Hh Protein inhibitors. Targeting of Hh pathway by Robotnikinin shows preventive role in metastasis and tumor relapse. GDC-0449 (Genentech), a small molecule Smo inhibitor, is used in treatment of BCC, colorectal cancer, ovarian cancer and other malignancies [147]. In a study it is shown that melatonin has beneficial effects in embryonic development through the hedgehog pathway [148].

## miRNA

MicroRNAs (miRNAs) are 21–25 nucleotide single strand non-coding RNAs and function as a part of post-transcriptional expression regulation system [149]. Multiple proteins including DICER work in collaboration to trim the primary RNA into the mature and functional form, miRNA. Downregulation of DICER has been reported in many cancers [150]. After binding with the compatible sequence of mRNA, they repress the translation of the mRNA by RNA-induced silencing complexes (RISC) with 3 main mechanisms: interrupting ribosome and mRNA interaction, cleavage of the mRNA into two non-functional segments (slicer-dependent mRNA degradation) and shortening the poly-A tail thus, destabilizing the mRNA. miRNAs have crucial effects on the regulation of cell proliferation, apoptosis, invasion, EMT and angiogenesis. Dysregulation in the expression of miRNAs are seen in many cancers suggesting their tumor suppressor or oncogene roles. Thus, they can be targeted or be used as biomarkers [151]. For example, downregulation of miRNA-145 augments the proliferation, invasion and metastasis of colon adenocarcinoma through over-activation of MAPK-1 [152]. TGFβ signaling pathway can alter the expression of miRNAs in prostate, breast and ovarian cancers and accelerate the metastatic progression of these malignancies [153]. Some aberrancies in the plasma levels of miRNAs (e.g. miRNA 196b, 198, 492, 614) are specific to pancreatic cancers and can be used as screening tests for detection of early cancer. miRNA-126 acts as a tumor suppressor and miRNA-197 as an oncogene leading to EMT [154]. Anti-sense oligonucleotides are used to target the up-regulated oncogene miRNAs reinforcing the main therapeutic regimen (chemosensitization) or even altering the phenotype of malignant cells into a more responsive type [153, 155]. Some of these new therapeutics have undergone human clinical trials. For example, OGX011 has been evaluated in metastatic prostate and breast cancers but minimal efficacy was benefited [156, 157]. miRNAs are also applied to predict the response to treat and individualizing the treatment of prostate, breast and ovarian cancers.

Recent in vivo studies show that administering melatonin to cancer cell lines alters miRNA expression and in that regard can be used as an anti-cancer agent [158]. Incubation of prostate cancer cells with melatonin upregulated miRNA 3195 and 374b expression, suppressing angiogenesis [159]. Melatonin also reduces the cytoplasmic levels of miRNA-24 by increasing its degradation after transcription. The mentioned miRNA targets p38 and p53 and is upregulated in colon, breast and head & neck cancers [160]. By downregulating oncogene miRNA-155, melatonin reduces invasion and proliferation of glioma [161]. Melatonin regulates miR-16-5p-Smad3 pathway reducing gastric cancer cell proliferation [162].

## Long non-coding RNAs

Defined as transcripts of more than 200 nucleotides which do not code any proteins, long non-coding RNAs (LncRNAs) were initially labeled cellular junk, but more investigation showed that they have an indisputable role in the regulation of gene expression, thus in regulating human pathologies [163]. Studying in vivo models of cancer cells showed that LncRNAs were indeed one of the possible targets for therapy in cancer. A set of acclaimed studies found that a balance between LncRNAs such as MALAT1, HOTAIR, PTV1 which favor epithelial mesenchymal transformation and LincRNA-p21 and NEAT1 which favor mesenchymal epithelial transformation balanced the aggressiveness of cancer cells in the tumor microenvironment, making the aforementioned LncRNAs possible targets for therapy [164]. Furthermore, analysis of random cancer cell lines of multiple neoplasias show that a bundle of LncRNAs are upregulated or downregulated, showing a possible etiologic role for these LncRNAs [165]. More so, LncRNA are shown to be associated with hallmarks of cancer such as evading apoptosis, resisting the immune response and altered cellular metabolism, making them a target for rendering cancers more susceptible for treatment, and changing the phenotype of cancer cells to more subtle phenotypes [166].

Theoretically, it is anticipated that there will be considerable overlap between the pathways melatonin effects and those that LncRNAs regulate. Recent studies have shown some of these interactions. Wang et al. showed that administration of melatonin to hepatocellular carcinoma cell lines significantly inhibited cellular proliferation and increased the expression of FOXA2, a transcription factor. Some of the anti-cancer effects of melatonin were mediated by LncRNA-CPS1-IT which inactivated HIF-1 alpha, an agent which is involved in the process of angiogenesis. This was accompanied by reduced rates of EMT, showing a reduced tendency towards invasion and metastasis [167]. Chen et al. found that LncRNA RAD51-ASI was able to sensitize hepatocellular carcinoma cells to conventional therapy agents, by inhibiting the translation of RAD51 micro RNA, a molecule involved in sensation of DNA damage, thus corrupting the process of DNA damage response, and resulting in inhibited DNA repair [168].

## Clinical significance of utilizing melatonin as a therapeutic agent

Melatonin is available in multiple drug forms, with favorable pharmacokinetic characteristics, and with no serious side effects [169]. Currently, multiple trials have shown the efficacy of melatonin in reducing the symptoms of conditions such as jet lag, irritable bowel syndrome, glaucoma, macular degeneration, hypertension and diabetes. It is also thought to be beneficial in targeting multiple malignancies, and in reducing the side effects of chemotherapy and radiation therapy [170]. Studies also suggest a possible role for exogenous melatonin in the treatment of pediatric sleep disorders, infectious disease, attention deficit hyperactivity disorders, epilepsy being the most important [171]. There is also a considerable body of evidence suggesting its use in neurodegenerative disorders and stroke [172]. Melatonin is both used as an adjuvant therapy and also as single therapy to target these conditions, as it is capable of directly affecting the function of signaling pathways which play etiologic roles in these conditions. Examples are discussed in detail in previous paragraphs, and the same could theoretically be true in human subjects. It is now well understood that diseases previously labeled as un-treatable are resulted from uncontrolled activation of the aforementioned pathways, such as increased inflammatory response in multiple sclerosis, increased grow factor signaling in neoplasms, and dopamine signaling pathways in mood disorders and schizophrenia [173, 174]. With increased understanding of the role of signaling pathways in human pathologies, more possibilities will emerge for the use of melatonin in clinical contexts. Currently, some hurdles exist before melatonin can gain wide clinical usage. The main issue is regarding the optimal dosage of melatonin for various conditions. In in vitro and in vivo studies, varying concentrations of melatonin have been used, sometimes being hundreds of times apart. As melatonin is being considered to be prescribed to individuals with varying conditions, a wide range of doses should be given. No large-scale clinical trial addresses this issue until now. Another issue is formulating suitable regimens in which melatonin is prescribed with other medication. Many possible interactions may exist between melatonin and other medications, which could limit its clinical use. More so, many combinations may be possible, and more specified trials will be needed to guide clinical decision making.

## Conclusion

Melatonin is a molecule with multiple functional roles, which is involved in important cellular processes. Melatonin affects cell signaling pathways both by receptor dependent and independent mechanisms. As mentioned before, the signaling pathways which melatonin affects are involved in much human pathology, ranging from cancer, to neurodegenerative diseases, and other subtler conditions, such as irritable bowel syndrome and sleep problems. Numerous in vivo and in vitro studies have outlined the exact mechanism of melatonin’s beneficial effect on these conditions, and more accumulating evidence suggests that melatonin should be used in clinical contexts. This is especially accurate for cancers. currently, many regimens exist for numerous malignancies, and melatonin could be a suitable addition to these regimens, both acting as an anti-cancer agent, which limits cellular proliferation, promotes apoptosis, counters cancer cell metabolism changes, reduces cellular migration and limiting metastasis, and also acting as adjuvant therapy, increasing quality of life in these cancer patients, and reducing the side effects of treatments.

## Data Availability

Not applicable.

## Abbreviations

CSC: Cancer stem cells
DDR: DNA damage response
EGCG: Epigallocatechin gallate
FDA: Food and Drug Administration
FGF: Fibroblast growth factor
GSIs: γ-Secretase inhibitors
GSCs: Glioblastoma stem-like cells
GISTs: Gastrointestinal stromal tumors
Hh: Hedgehog
IAPs: Inhibitors of apoptotic proteins
lncRNAs: Long non-coding RNAs
MMP: Metalloproteinase
miRNAs: MicroRNAs
mTOR: Mammalian target of rapamycin
PDK1: 3-Phosphoinositide-dependent kinase 1
PDGF: Platelet-derived growth factor
PI2P: Phosphatidylinositol 4,5 diphosphate
PTEN: Phosphatase and tensin homologue deleted on chromosome
ROS: Reactive oxygen species
ROCK: Rho-dependent protein kinase
RISC: RNA-induced silencing complexes
UPR: Unfolded protein response
VEGF: Vascular endothelial growth factor

## References

[1] Florido J, Rodriguez-Santana C, Martinez-Ruiz L, López-Rodríguez A, Acuña-Castroviejo D, Rusanova I (2022). Understanding the mechanism of action of melatonin, which induces ROS production in cancer cells. Antioxidants.

[2] Tan DX, Hardeland R, Manchester LC, Paredes SD, Korkmaz A, Sainz RM (2010). The changing biological roles of melatonin during evolution: from an antioxidant to signals of darkness, sexual selection and fitness. Biol Rev.

[3] Samanta S (2020). Melatonin: an endogenous miraculous indolamine, fights against cancer progression. J Cancer Res Clin Oncol.

[4] Talib WH, Alsayed AR, Abuawad A, Daoud S, Mahmod AI (2021). Melatonin in cancer treatment: current knowledge and future opportunities. Molecules.

[5] Martin GS (2003). Cell signaling and cancer. Cancer Cell.

[6] Bonmati-Carrion M-A, Tomas-Loba A (2021). Melatonin and cancer: a polyhedral network where the source matters. Antioxidants.

[7] Hanahan D (2022). Hallmarks of cancer: new dimensions. Cancer Discov.

[8] Núñez Martínez P, Zapico S. Melatonin: a double-edged sword for cancer treatment. Free Radicals and Health. 2016.

[9] Di Bella G, Mascia F, Gualano L, Di Bella L (2013). Melatonin anticancer effects: review. Int J Mol Sci.

[10] Srinivasan V, Pandi-Perumal SR, Brzezinski A, Bhatnagar KP, Cardinali DP (2011). Melatonin, immune function and cancer. Recent Patents Endocr Metab Immune Drug Discov..

[11] Salvatore V, Teti G, Focaroli S, Mazzotti MC, Mazzotti A, Falconi M (2016). The tumor microenvironment promotes cancer progression and cell migration. Oncotarget.

[12] Sonehara NM, Lacerda JZ, Jardim-Perassi BV, de Paula JR, Moschetta-Pinheiro MG, Souza YST (2019). Melatonin regulates tumor aggressiveness under acidosis condition in breast cancer cell lines. Oncol Lett.

[13] Majidinia M, Sadeghpour A, Mehrzadi S, Reiter RJ, Khatami N, Yousefi B (2017). Melatonin: a pleiotropic molecule that modulates DNA damage response and repair pathways. J Pineal Res.

[14] Majidinia M, Bishayee A, Yousefi B (2019). Polyphenols: major regulators of key components of DNA damage response in cancer. DNA Repair.

[15] Lin C, McGough R, Aswad B, Block JA, Terek R (2004). Hypoxia induces HIF-1alpha and VEGF expression in chondrosarcoma cells and chondrocytes. J Orthop Res.

[16] Rajabi M, Mousa SA (2017). The role of angiogenesis in cancer treatment. Biomedicines.

[17] Goradel NH, Asghari MH, Moloudizargari M, Negahdari B, Haghi-Aminjan H, Abdollahi M (2017). Melatonin as an angiogenesis inhibitor to combat cancer: Mechanistic evidence. Toxicol Appl Pharmacol.

[18] Menndez-Menndez J, Mart, et al. Melatonin: an anti-tumor agent in hormone-dependent cancers. Int J Endocrinol. 2018;2018:20. 10.1155/2018/3271948 PMC6189685 30386380

[19] Ring A, Dowsett M (2004). Mechanisms of tamoxifen resistance. Endocr Relat Cancer.

[20] Chen WY, Giobbie-Hurder A, Gantman K, Savoie J, Scheib R, Parker LM (2014). A randomized, placebo-controlled trial of melatonin on breast cancer survivors: impact on sleep, mood, and hot flashes. Breast Cancer Res Treat.

[21] Yousefi B, Azimi A, Majidinia M, Shafiei-Irannejad V, Badalzadeh R, Baradaran B (2017). Balaglitazone reverses P-glycoprotein-mediated multidrug resistance via upregulation of PTEN in a PPARγ-dependent manner in leukemia cells. Tumor Biology.

[22] Song G, Ouyang G, Bao S (2005). The activation of Akt/PKB signaling pathway and cell survival. J Cell Mol Med.

[23] Baek SH, Ko JH, Lee JH, Kim C, Lee H, Nam D (2017). Ginkgolic acid inhibits invasion and migration and TGF-β-induced EMT of lung cancer cells through PI3K/Akt/mTOR inactivation. J Cell Physiol.

[24] Jiao D, Wang J, Lu W, Tang X, Chen J, Mou H (2016). Curcumin inhibited HGF-induced EMT and angiogenesis through regulating c-Met dependent PI3K/Akt/mTOR signaling pathways in lung cancer. Mol Therapy-Oncolytics.

[25] Chen B, Li D, Li M, Li S, Peng K, Shi X (2016). Induction of mitochondria-mediated apoptosis and PI3K/Akt/ mTOR-mediated autophagy by aflatoxin B2 in hepatocytes of broilers. Oncotarget.

[26] Yang J, Pi C, Wang G (2018). Inhibition of PI3K/Akt/mTOR pathway by apigenin induces apoptosis and autophagy in hepatocellular carcinoma cells. Biomed Pharmacother.

[27] Sasore T, Reynolds AL, Kennedy BN (2014). Targeting the PI3K/Akt/mTOR pathway in ocular neovascularization. Adv Exp Med Biol.

[28] Jacot JL, Sherris D (2011). Potential therapeutic roles for inhibition of the PI3K/Akt/mTOR pathway in the pathophysiology of diabetic retinopathy. J Ophthalmol.

[29] Gao N, Zhang Z, Jiang B-H, Shi X (2003). Role of PI3K/AKT/mTOR signaling in the cell cycle progression of human prostate cancer. Biochem Biophys Res Commun.

[30] Fekete M, Santiskulvong C, Eng C, Dorigo O (2012). Effect of PI3K/Akt pathway inhibition-mediated G1 arrest on chemosensitization in ovarian cancer cells. Anticancer Res.

[31] Mao L, Dauchy RT, Blask DE, Dauchy EM, Slakey LM, Brimer S (2016). Melatonin suppression of aerobic glycolysis (Warburg effect), survival signalling and metastasis in human leiomyosarcoma. J Pineal Res.

[32] Carracedo A, Ma L, Teruya-Feldstein J, Rojo F, Salmena L, Alimonti A (2008). Inhibition of mTORC1 leads to MAPK pathway activation through a PI3K-dependent feedback loop in human cancer. J Clin Investig.

[33] Castellano E, Downward J (2011). RAS interaction with PI3K: more than just another effector pathway. Genes Cancer.

[34] Prieto-Domínguez N, Méndez-Blanco C, Carbajo-Pescador S, Fondevila F, García-Palomo A, González-Gallego J (2017). Melatonin enhances sorafenib actions in human hepatocarcinoma cells by inhibiting mTORC1/p70S6K/HIF-1α and hypoxia-mediated mitophagy. Oncotarget.

[35] Liu C, Jia Z, Zhang X, Hou J, Wang L, Hao S (2012). Involvement of melatonin in autophagy-mediated mouse hepatoma H22 cell survival. Int Immunopharmacol.

[36] McCubrey JA, Steelman LS, Chappell WH, Abrams SL, Montalto G, Cervello M (2012). Mutations and deregulation of Ras/Raf/MEK/ERK and PI3K/PTEN/Akt/mTOR cascades which alter therapy response. Oncotarget.

[37] Shen Y-Q, Guerra-Librero A, Fernandez-Gil BI, Florido J, García-López S, Martinez-Ruiz L (2018). Combination of melatonin and rapamycin for head and neck cancer therapy: suppression of AKT/mTOR pathway activation, and activation of mitophagy and apoptosis via mitochondrial function regulation. J Pineal Res.

[38] Fernández A, Ordóñez R, Reiter RJ, González-Gallego J, Mauriz JL (2015). Melatonin and endoplasmic reticulum stress: relation to autophagy and apoptosis. J Pineal Res.

[39] Diehl JA, Fuchs SY, Koumenis C (2011). The cell biology of the unfolded protein response. Gastroenterology.

[40] Park SW, Ozcan U (2013). Potential for therapeutic manipulation of the UPR in disease. Semin Immunopathol.

[41] Bizzarri M, Proietti S, Cucina A, Reiter RJ (2013). Molecular mechanisms of the pro-apoptotic actions of melatonin in cancer: a review. Expert Opin Ther Targets.

[42] Sainz RM, Mayo JC, Rodriguez C, Tan DX, Lopez-Burillo S, Reiter RJ (2003). Melatonin and cell death: differential actions on apoptosis in normal and cancer cells. Cell Mol Life Sci CMLS.

[43] Jou MJ, Peng TI, Reiter RJ, Jou SB, Wu HY, Wen ST (2004). Visualization of the antioxidative effects of melatonin at the mitochondrial level during oxidative stress-induced apoptosis of rat brain astrocytes. J Pineal Res.

[44] Rodriguez C, Martín V, Herrera F, García-Santos G, Rodriguez-Blanco J, Casado-Zapico S (2013). Mechanisms involved in the pro-apoptotic effect of melatonin in cancer cells. Int J Mol Sci.

[45] Kimball SR, Abbas A, Jefferson LS (2008). Melatonin represses oxidative stress-induced activation of the MAP kinase and mTOR signaling pathways in H4IIE hepatoma cells through inhibition of Ras. J Pineal Res.

[46] Kim HS, Kim T-J, Yoo Y-M (2014). Melatonin combined with endoplasmic reticulum stress induces cell death via the PI3K/Akt/mTOR pathway in B16F10 melanoma cells. PLoS ONE.

[47] Ferreira GM, Martinez M, Camargo ICC, Domeniconi RF, Martinez FE, Chuffa LGA (2014). Melatonin attenuates Her-2, p38 MAPK, p-AKT, and mTOR levels in ovarian carcinoma of ethanol-preferring rats. J Cancer.

[48] Martín V, Herrera F, Carrera-Gonzalez P, García-Santos G, Antolín I, Rodriguez-Blanco J (2006). Intracellular signaling pathways involved in the cell growth inhibition of glioma cells by melatonin. Can Res.

[49] Reiter RJ, Tan DX, Sainz RM, Mayo JC, Lopez-Burillo S (2002). Melatonin: reducing the toxicity and increasing the efficacy of drugs. J Pharm Pharmacol.

[50] Girish KS, Paul M, Thushara RM, Hemshekhar M, Shanmuga Sundaram M, Rangappa KS (2013). Melatonin elevates apoptosis in human platelets via ROS mediated mitochondrial damage. Biochem Biophys Res Commun.

[51] Fecher LA, Amaravadi RK, Flaherty KT (2008). The MAPK pathway in melanoma. Curr Opin Oncol.

[52] Molina JR, Adjei AA (2006). The Ras/Raf/MAPK pathway. J Thorac Oncol.

[53] Martin KC, Michael D, Rose JC, Barad M, Casadio A, Zhu H (1997). MAP kinase translocates into the nucleus of the presynaptic cell and is required for long-term facilitation in aplysia. Neuron.

[54] Plotnikov A, Zehorai E, Procaccia S, Seger R (2011). The MAPK cascades: signaling components, nuclear roles and mechanisms of nuclear translocation. Biochim Biophys Acta (BBA) Mol Cell Res..

[55] Roy SK, Srivastava RK, Shankar S (2010). Inhibition of PI3K/AKT and MAPK/ERK pathways causes activation of FOXO transcription factor, leading to cell cycle arrest and apoptosis in pancreatic cancer. J Mol Signaling.

[56] Fang JY, Richardson BC (2005). The MAPK signalling pathways and colorectal cancer. Lancet Oncol.

[57] Anjum J, Mitra S, Das R, Alam R, Mojumder A, Emran TB (2022). A renewed concept on the MAPK signaling pathway in cancers: polyphenols as a choice of therapeutics. Pharmacol Res.

[58] Mendoza MC, Er EE, Blenis J (2011). The Ras-ERK and PI3K-mTOR pathways: cross-talk and compensation. Trends Biochem Sci.

[59] Stefani C, Miricescu D, Stanescu-Spinu I-I, Nica RI, Greabu M, Totan AR (2021). Growth factors, PI3K/AKT/mTOR and MAPK signaling pathways in colorectal cancer pathogenesis: where are we now?. Int J Mol Sci.

[60] Zohrabian VM, Forzani B, Chau Z, Murali R, Jhanwar-Uniyal M (2009). Rho/ROCK and MAPK signaling pathways are involved in glioblastoma cell migration and proliferation. Anticancer Res.

[61] Hill SM, Belancio VP, Dauchy RT, Xiang S, Brimer S, Mao L (2015). Melatonin: an inhibitor of breast cancer. Endocr Relat Cancer.

[62] Mao L, Yuan L, Slakey LM, Jones FE, Burow ME, Hill SM (2010). Inhibition of breast cancer cell invasion by melatonin is mediated through regulation of the p38 mitogen-activated protein kinase signaling pathway. Breast Cancer Res.

[63] Slakey LM, Frasch T, Blask DE, Dauchy EM, Dauchy RT, Yuan L (2012). Circadian gating of epithelial-to-mesenchymal transition in breast cancer cells via melatonin-regulation of GSK3β. Mol Endocrinol.

[64] Dauchy RT, Xiang S, Mao L, Brimer S, Wren MA, Yuan L (2014). Circadian and melatonin disruption by exposure to light at night drives intrinsic resistance to tamoxifen therapy in breast cancer. Cancer Res.

[65] Joo SS, Yoo Y-M (2009). Melatonin induces apoptotic death in LNCaP cells via p38 and JNK pathways: therapeutic implications for prostate cancer. J Pineal Res.

[66] Carbajo-Pescador S, García-Palomo A, Martín-Renedo J, Piva M, González-Gallego J, Mauriz JL (2011). Melatonin modulation of intracellular signaling pathways in hepatocarcinoma HepG2 cell line: role of the MT1 receptor. J Pineal Res.

[67] Zhou Q, Gui S, Zhou Q, Wang Y (2014). Melatonin inhibits the migration of human lung adenocarcinoma A549 cell lines involving JNK/MAPK pathway. PLoS ONE.

[68] Cabrera J, Negrín G, Estévez F, Loro J, Reiter RJ, Quintana J (2010). Melatonin decreases cell proliferation and induces melanogenesis in human melanoma SK-MEL-1 cells. J Pineal Res.

[69] Li W, Fan M, Chen Y, Zhao Q, Song C, Yan Y (2015). Melatonin induces cell apoptosis in AGS cells through the activation of JNK and P38 MAPK and the suppression of nuclear factor-kappa B: a novel therapeutic implication for gastric cancer. Cell Physiol Biochem.

[70] Ho H-Y, Lin C-W, Chien M-H, Reiter RJ, Su S-C, Hsieh Y-H (2016). Melatonin suppresses TPA-induced metastasis by downregulating matrix metalloproteinase-9 expression through JNK/SP-1 signaling in nasopharyngeal carcinoma. J Pineal Res.

[71] Yeh C-M, Lin C-W, Yang J-S, Yang W-E, Su S-C, Yang S-F (2016). Melatonin inhibits TPA-induced oral cancer cell migration by suppressing matrix metalloproteinase-9 activation through the histone acetylation. Oncotarget.

[72] Ortíz-López L, Morales-Mulia S, Ramírez-Rodríguez G, Benítez-King G (2009). ROCK-regulated cytoskeletal dynamics participate in the inhibitory effect of melatonin on cancer cell migration. J Pineal Res.

[73] Bikkavilli RK, Malbon CC (2009). Mitogen-activated protein kinases and Wnt/β-catenin signaling: molecular conversations among signaling pathways. Commun Integr Biol.

[74] Lorzadeh S, Kohan L, Ghavami S, Azarpira N (2021). Autophagy and the Wnt signaling pathway: A focus on Wnt/β-catenin signaling. Biochim Biophys Acta (BBA)-Mol Cell Res.

[75] Aberle H, Bauer A, Stappert J, Kispert A, Kemler R (1997). β-catenin is a target for the ubiquitin–proteasome pathway. EMBO J.

[76] Kohn AD, Moon RT (2005). Wnt and calcium signaling: β-Catenin-independent pathways. Cell Calcium.

[77] Fodde R, Brabletz T (2007). Wnt/β-catenin signaling in cancer stemness and malignant behavior. Curr Opin Cell Biol.

[78] Chen B, Dodge ME, Tang W, Lu J, Ma Z, Fan C-W (2009). Small molecule-mediated disruption of Wnt-dependent signaling in tissue regeneration and cancer. Nat Chem Biol.

[79] Fu Y, Chang H, Peng X, Bai Q, Yi L, Zhou Y (2014). Resveratrol inhibits breast cancer stem-like cells and induces autophagy via suppressing Wnt/β-catenin signaling pathway. PLoS ONE.

[80] Matsuda Y, Schlange T, Oakeley EJ, Boulay A, Hynes NE (2009). WNT signaling enhances breast cancer cell motility and blockade of the WNT pathway by sFRP1 suppresses MDA-MB-231 xenograft growth. Breast Cancer Res.

[81] Anastas JN, Moon RT (2012). WNT signalling pathways as therapeutic targets in cancer. Nat Rev Cancer.

[82] Takahashi-Yanaga F, Kahn M (2010). Targeting Wnt signaling: can we safely eradicate cancer stem cells?. Clin Cancer Res.

[83] Barker N, Clevers H (2006). Mining the Wnt pathway for cancer therapeutics. Nat Rev Drug Discov.

[84] Liu S, Liang B, Jia H, Jiao Y, Pang Z, Huang Y (2017). Evaluation of cell death pathways initiated by antitumor drugs melatonin and valproic acid in bladder cancer cells. FEBS Open Bio.

[85] Gravdal K, Halvorsen OJ, Haukaas SA, Akslen LA (2007). A switch from E-cadherin to N-cadherin expression indicates epithelial to mesenchymal transition and is of strong and independent importance for the progress of prostate cancer. Clin Cancer Res.

[86] Zhang J, Xie T, Zhong X, Jiang H-L, Li R, Wang B-Y (2020). Melatonin reverses nasopharyngeal carcinoma cisplatin chemoresistance by inhibiting the Wnt/β-catenin signaling pathway. Aging (Albany NY).

[87] Bu S, Wang Q, Sun J, Li X, Gu T, Lai D (2020). Melatonin suppresses chronic restraint stress-mediated metastasis of epithelial ovarian cancer via NE/AKT/β-catenin/SLUG axis. Cell Death Dis.

[88] Li Y, Zou J, Li B, Du J (2021). Anticancer effects of melatonin via regulating lncRNA JPX-Wnt/β-catenin signalling pathway in human osteosarcoma cells. J Cell Mol Med.

[89] Zhou B, Lin W, Long Y, Yang Y, Zhang H, Wu K (2022). Notch signaling pathway: architecture, disease, and therapeutics. Signal Transduct Target Ther.

[90] Guo S, Liu M, Gonzalez-Perez RR (2011). Role of Notch and its oncogenic signaling crosstalk in breast cancer. Biochim Biophys Acta (BBA) Rev Cancer..

[91] Villaronga M, Bevan CL, Belandia B (2008). Notch signaling: a potential therapeutic target in prostate cancer. Curr Cancer Drug Targets.

[92] Aster JC (2005). Deregulated NOTCH signaling in acute T-cell lymphoblastic leukemia/lymphoma: new insights, questions, and opportunities. Int J Hematol.

[93] Real PJ, Tosello V, Palomero T, Castillo M, Hernando E, De Stanchina E (2009). γ-secretase inhibitors reverse glucocorticoid resistance in T cell acute lymphoblastic leukemia. Nat Med.

[94] Rizzo P, Osipo C, Foreman K, Golde T, Osborne B, Miele L (2008). Rational targeting of Notch signaling in cancer. Oncogene.

[95] Peters J-U, Galley G, Jacobsen H, Czech C, David-Pierson P, Kitas EA (2007). Novel orally active, dibenzazepinone-based γ-secretase inhibitors. Bioorg Med Chem Lett.

[96] Hoey T, Yen W-C, Axelrod F, Basi J, Donigian L, Dylla S (2009). DLL4 blockade inhibits tumor growth and reduces tumor-initiating cell frequency. Cell Stem Cell.

[97] Wu Y, Cain-Hom C, Choy L, Hagenbeek TJ, de Leon GP, Chen Y (2010). Therapeutic antibody targeting of individual Notch receptors. Nature.

[98] Zheng X, Pang B, Gu G, Gao T, Zhang R, Pang Q (2017). Melatonin inhibits glioblastoma stem-like cells through suppression of EZH2-NOTCH1 signaling axis. Int J Biol Sci.

[99] Margheri M, Pacini N, Tani A, Nosi D, Squecco R, Dama A (2012). Combined effects of melatonin and all-trans retinoic acid and somatostatin on breast cancer cell proliferation and death: molecular basis for the anticancer effect of these molecules. Eur J Pharmacol.

[100] Qi S, Yan L, Liu Z, Mu Y-L, Li M, Zhao X (2018). Melatonin inhibits 17β-estradiol-induced migration, invasion and epithelial-mesenchymal transition in normal and endometriotic endometrial epithelial cells. Reprod Biol Endocrinol.

[101] Zhu X, Chen S, Jiang Y, Xu Y, Zhao Y, Chen L (2018). Analysis of miRNA expression profiles in melatonin-exposed GC-1 spg cell line. Gene.

[102] Zinatizadeh MR, Schock B, Chalbatani GM, Zarandi PK, Jalali SA, Miri SR (2021). The Nuclear Factor Kappa B (NF-kB) signaling in cancer development and immune diseases. Genes Dis.

[103] Pasparakis M, Luedde T, Schmidt-Supprian M (2006). Dissection of the NF-κB signalling cascade in transgenic and knockout mice. Cell Death Differ.

[104] Yousefi B, Zarghami N, Samadi N, Majidinia M (2016). Peroxisome proliferator-activated receptors and their ligands in cancer drug-resistance: opportunity or challenge. Anti-Cancer Agents Med Chem (Formerly Current Medicinal Chemistry-Anti-Cancer Agents)..

[105] Yousefi B, Samadi N, Baradaran B, Rameshknia V, Shafiei-Irannejad V, Majidinia M (2015). Differential effects of peroxisome proliferator-activated receptor agonists on doxorubicin-resistant human myelogenous leukemia (K562/DOX) cells. Cell Mol Biol (Noisy-le-grand).

[106] Song L, Peng Y, Yun J, Rizzo P, Chaturvedi V, Weijzen S (2008). Notch-1 associates with IKKα and regulates IKK activity in cervical cancer cells. Oncogene.

[107] Sasaki N, Morisaki T, Hashizume K, Yao T, Tsuneyoshi M, Noshiro H (2001). Nuclear factor-κB p65 (RelA) transcription factor is constitutively activated in human gastric carcinoma tissue. Clin Cancer Res.

[108] Dong QG, Sclabas GM, Fujioka S, Schmidt C, Peng B, Wu T (2002). The function of multiple IκB: NF-κB complexes in the resistance of cancer cells to Taxol-induced apoptosis. Oncogene.

[109] Adams J, Palombella VJ, Sausville EA, Johnson J, Destree A, Lazarus DD (1999). Proteasome inhibitors: a novel class of potent and effective antitumor agents. Cancer Res.

[110] Véquaud E, Séveno C, Loussouarn D, Engelhart L, Campone M, Juin P (2015). YM155 potently triggers cell death in breast cancer cells through an autophagy-NF-kB network. Oncotarget.

[111] Charbonneau B, Block MS, Bamlet WR, Vierkant RA, Kalli KR, Fogarty Z (2014). Risk of ovarian cancer and the NF-κB pathway: genetic association with IL1A and TNFSF10. Cancer Res.

[112] Amaravadi RK, Schilder RJ, Martin LP, Levin M, Graham MA, Weng DE (2015). A phase I study of the SMAC-mimetic birinapant in adults with refractory solid tumors or lymphoma. Mol Cancer Ther.

[113] Mosieniak G, Adamowicz M, Alster O, Jaskowiak H, Szczepankiewicz AA, Wilczynski GM (2012). Curcumin induces permanent growth arrest of human colon cancer cells: link between senescence and autophagy. Mech Ageing Dev.

[114] Shishodia S, Aggarwal BB (2004). Nuclear factor-κB: a friend or a foe in cancer?. Biochem Pharmacol.

[115] Mohan N, Sadeghi K, Reiter RJ, Meltz ML (1995). The neurohormone melatonin inhibits cytokine, mitogen and ionizing radiation induced NF-kappa B. Biochem Mol Biol Int.

[116] Qin W, Lu W, Li H, Yuan X, Li B, Zhang Q (2012). Melatonin inhibits IL1β-induced MMP9 expression and activity in human umbilical vein endothelial cells by suppressing NF-κB activation. J Endocrinol.

[117] Colombo J, Jardim-Perassi BV, Ferreira JP, Braga CZ, Sonehara NM, Júnior RP (2018). Melatonin differentially modulates NF-кB expression in breast and liver cancer cells. Anti-Cancer Agents Med Chem (Formerly Current Medicinal Chemistry-Anti-Cancer Agents)..

[118] Markus R, Cecon E, Pires-Lapa M (2013). Immune-pineal axis: nuclear factor κB (NF-kB) mediates the shift in the melatonin source from pinealocytes to immune competent cells. Int J Mol Sci.

[119] Bromberg J (2002). Stat proteins and oncogenesis. J Clin Investig.

[120] O'Shea JJ, Gadina M, Schreiber RD (2002). Cytokine signaling in 2002: new surprises in the Jak/Stat pathway. Cell.

[121] Tang S-N, Fu J, Shankar S, Srivastava RK (2012). EGCG enhances the therapeutic potential of gemcitabine and CP690550 by inhibiting STAT3 signaling pathway in human pancreatic cancer. PLoS ONE.

[122] Masuda M, Suzui M, Lim JT, Weinstein IB (2003). Epigallocatechin-3-gallate inhibits activation of HER-2/neu and downstream signaling pathways in human head and neck and breast carcinoma cells. Clin Cancer Res.

[123] Shi X, Franko B, Frantz C, Amin HM, Lai R (2006). JSI-124 (cucurbitacin I) inhibits Janus kinase-3/signal transducer and activator of transcription-3 signalling, downregulates nucleophosmin-anaplastic lymphoma kinase (ALK), and induces apoptosis in ALK-positive anaplastic large cell lymphoma cells. Br J Haematol.

[124] Su Y, Li G, Zhang X, Gu J, Zhang C, Tian Z (2008). JSI-124 inhibits glioblastoma multiforme cell proliferation through G2/M cell cycle arrest and apoptosis augmentation. Cancer Biol Ther.

[125] Thoennissen NH, Iwanski GB, Doan NB, Okamoto R, Lin P, Abbassi S (2009). Cucurbitacin B induces apoptosis by inhibition of the JAK/STAT pathway and potentiates antiproliferative effects of gemcitabine on pancreatic cancer cells. Can Res.

[126] Xia Y, Chen S, Zeng S, Zhao Y, Zhu C, Deng B (2019). Melatonin in macrophage biology: current understanding and future perspectives. J Pineal Res.

[127] Ji ZZ, Xu YC (2016). Melatonin protects podocytes from angiotensin II-induced injury in an in vitro diabetic nephropathy model. Mol Med Rep.

[128] Wang T, Dong Y, Wang Z, Cao J, Chen Y (2016). Secretion pathway of liver IGF-1 via JAK2/STAT3 in chick embryo under the monochromatic light. Growth Factors.

[129] Buendia I, Gomez-Rangel V, Gonzalez-Lafuente L, Parada E, Leon R, Gameiro I (2015). Neuroprotective mechanism of the novel melatonin derivative Neu-P11 in brain ischemia related models. Neuropharmacology.

[130] Qiu J, Zhang J, Zhou Y, Li X, Li H, Liu J (2019). MicroRNA-7 inhibits melatonin synthesis by acting as a linking molecule between leptin and norepinephrine signaling pathways in pig pineal gland. J Pineal Res.

[131] Vincent AM, Feldman EL (2002). Control of cell survival by IGF signaling pathways. Growth Hormon IGF Res.

[132] Cao Y, Cao R, Hedlund E-M (2008). R regulation of tumor angiogenesis and metastasis by FGF and PDGF signaling pathways. J Mol Med.

[133] Katoh M (2007). Networking of WNT, FGF, Notch, BMP, and Hedgehog signaling pathways during carcinogenesis. Stem Cell Rev.

[134] Brooks AN, Kilgour E, Smith PD (2012). Molecular pathways: fibroblast growth factor signaling: a new therapeutic opportunity in cancer. Clin Cancer Res.

[135] Ferrara N, Hillan KJ, Gerber H-P, Novotny W (2004). Discovery and development of bevacizumab, an anti-VEGF antibody for treating cancer. Nat Rev Drug Discov.

[136] Gasparini G, Longo R, Toi M, Ferrara N (2005). Angiogenic inhibitors: a new therapeutic strategy in oncology. Nat Rev Clin Oncol.

[137] Jain RK, Duda DG, Clark JW, Loeffler JS (2006). Lessons from phase III clinical trials on anti-VEGF therapy for cancer. Nat Rev Clin Oncol.

[138] Wu Y, Hooper AT, Zhong Z, Witte L, Bohlen P, Rafii S (2006). The vascular endothelial growth factor receptor (VEGFR-1) supports growth and survival of human breast carcinoma. Int J Cancer.

[139] Lovly CM, McDonald NT, Chen H, Ortiz-Cuaran S, Heukamp LC, Yan Y (2014). Rationale for co-targeting IGF-1R and ALK in ALK fusion-positive lung cancer. Nat Med.

[140] Chen HX, Sharon E (2013). IGF-1R as an anti-cancer target—trials and tribulations. Chin J Cancer.

[141] Desnoyers L, Pai R, Ferrando R, Hötzel K, Le T, Ross J (2008). Targeting FGF19 inhibits tumor growth in colon cancer xenograft and FGF19 transgenic hepatocellular carcinoma models. Oncogene.

[142] Mayo JC, Hevia D, Quiros-Gonzalez I, Rodriguez-Garcia A, Gonzalez-Menendez P, Cepas V (2017). IGFBP 3 and MAPK/ERK signaling mediates melatonin-induced antitumor activity in prostate cancer. J Pineal Res.

[143] Gelaleti GB, Borin TF, Maschio-Signorini LB, Moschetta MG, Jardim-Perassi BV, Calvinho GB (2017). Efficacy of melatonin, IL-25 and siIL-17B in tumorigenesis-associated properties of breast cancer cell lines. Life Sci.

[144] Marques JH, Mota AL, Oliveira JG, Lacerda JZ, Stefani JP, Ferreira LC (2018). Melatonin restrains angiogenic factors in triple-negative breast cancer by targeting miR-152-3p: In vivo and in vitro studies. Life Sci.

[145] Micchelli CA, Selva E, Mogila V, Perrimon N (2002). Rasp, a putative transmembrane acyltransferase, is required for Hedgehog signaling. Development.

[146] Nguyen NM, Cho J (2022). Hedgehog pathway inhibitors as targeted cancer therapy and strategies to overcome drug resistance. Int J Mol Sci.

[147] Von Hoff DD, LoRusso PM, Rudin CM, Reddy JC, Yauch RL, Tibes R (2009). Inhibition of the hedgehog pathway in advanced basal-cell carcinoma. N Engl J Med.

[148] Lee S, Jin JX, Taweechaipaisankul A, Kim GA, Ahn C, Lee BC (2017). Melatonin influences the sonic hedgehog signaling pathway in porcine cumulus oocyte complexes. J Pineal Res.

[149] Moein S, Vaghari-Tabari M, Qujeq D, Majidinia M, Nabavi SM, Yousefi B (2019). MiRNAs and inflammatory bowel disease: an interesting new story. J Cell Physiol.

[150] Hill M, Tran N (2021). miRNA interplay: mechanisms and consequences in cancer. Dis Models Mech..

[151] Ji W, Sun B, Su C (2017). Targeting microRNAs in cancer gene therapy. Genes.

[152] Yang Y, Li X-J, Li P, Guo X-T (2018). MicroRNA-145 regulates the proliferation, migration and invasion of human primary colon adenocarcinoma cells by targeting MAPK1. Int J Mol Med.

[153] Smith B, Agarwal P, Bhowmick NA (2017). MicroRNA applications for prostate, ovarian and breast cancer in the era of precision medicine. Endocr Relat Cancer.

[154] Słotwiński R, Lech G, Słotwińska SM (2018). MicroRNAs in pancreatic cancer diagnosis and therapy. Central-Eur J Immunol.

[155] Stahlhut C, Slack FJ (2013). MicroRNAs and the cancer phenotype: profiling, signatures and clinical implications. Genome Med.

[156] Chia S, Dent S, Ellard S, Ellis PM, Vandenberg T, Gelmon K (2009). Phase II trial of OGX-011 in combination with docetaxel in metastatic breast cancer. Clin Cancer Res.

[157] Beer TM, Hotte SJ, Saad F, Alekseev B, Matveev V, Fléchon A (2017). Custirsen (OGX-011) combined with cabazitaxel and prednisone versus cabazitaxel and prednisone alone in patients with metastatic castration-resistant prostate cancer previously treated with docetaxel (AFFINITY): a randomised, open-label, international, phase 3 trial. Lancet Oncol.

[158] Lee SE, Kim SJ, Youn J-P, Hwang SY, Park C-S, Park YS (2011). MicroRNA and gene expression analysis of melatonin-exposed human breast cancer cell lines indicating involvement of the anticancer effect. J Pineal Res.

[159] Sohn EJ, Won G, Lee J, Lee S, Kim S-H (2015). Upregulation of miRNA3195 and miRNA374b mediates the anti-angiogenic properties of melatonin in hypoxic PC-3 prostate cancer cells. J Cancer.

[160] Mori F, Ferraiuolo M, Santoro R, Sacconi A, Goeman F, Pallocca M (2016). Multitargeting activity of miR-24 inhibits long-term melatonin anticancer effects. Oncotarget.

[161] Gu J, Lu Z, Ji C, Chen Y, Liu Y, Lei Z (2017). Melatonin inhibits proliferation and invasion via repression of miRNA-155 in glioma cells. Biomed Pharmacother.

[162] Chenyu Z, Qun H, Hongyu Z (2018). Melatonin inhibits the proliferation of gastric cancer cells through regulating the miR-16-5p-Smad3 pathway. DNA Cell Biol.

[163] Abolghasemi M, Tehrani SS, Yousefi T, Karimian A, Mahmoodpoor A, Ghamari A (2020). Critical roles of long noncoding RNAs in breast cancer. J Cell Physiol.

[164] Sanchez Calle A, Kawamura Y, Yamamoto Y, Takeshita F, Ochiya T (2018). Emerging roles of long non-coding RNA in cancer. Cancer Sci.

[165] Arun G, Diermeier SD, Spector DL (2018). Therapeutic targeting of long non-coding RNAs in cancer. Trends Mol Med.

[166] Parasramka MA, Maji S, Matsuda A, Yan IK, Patel T (2016). Long non-coding RNAs as novel targets for therapy in hepatocellular carcinoma. Pharmacol Ther.

[167] Wang TH, Wu CH, Yeh CT, Su SC, Hsia SM, Liang KH (2017). Melatonin suppresses hepatocellular carcinoma progression via lncRNA-CPS1-IT-mediated HIF-1alpha inactivation. Oncotarget.

[168] Chen C-C, Chen C-Y, Wang S-H, Yeh C-T, Su S-C, Ueng S-H (2018). Melatonin sensitizes hepatocellular carcinoma cells to chemotherapy through long non-coding RNA RAD51-AS1-mediated suppression of DNA repair. Cancers.

[169] Andersen LP, Gogenur I, Rosenberg J, Reiter RJ (2016). The safety of melatonin in humans. Clin Drug Investig.

[170] Sánchez-Barceló E, Mediavilla M, Tan D, Reiter R (2010). Clinical uses of melatonin: evaluation of human trials. Curr Med Chem.

[171] Sánchez-Barceló EJ, Mediavilla MD, Reiter RJ (2011). Clinical uses of melatonin in pediatrics. Int J Pediatr.

[172] Miller E, Morel A, Saso L, Saluk J (2015). Melatonin redox activity. Its potential clinical applications in neurodegenerative disorders. Curr Top Med Chem.

[173] Karam CS, Ballon JS, Bivens NM, Freyberg Z, Girgis RR, Lizardi-Ortiz JE (2010). Signaling pathways in schizophrenia: emerging targets and therapeutic strategies. Trends Pharmacol Sci.

[174] Sever R, Brugge JS (2015). Signal transduction in cancer. Cold Spring Harbor Perspect Med..

[175] Sánchez-Sánchez A, Antolin I, Puente-Moncada N, Suarez S, Gomez-Lobo M, Rodriguez C, et al. Melatonin cytotoxicity is associated to Warburg effect inhibition in ewing sarcoma cells. PLoS One. 2015; e0135420. 10.1371/journal.pone.0135420 PMC4529102 26252771

[176] Lin Y-W, Lee L-M, Lee W-J, Chu C-Y, Tan P, Yang Y-C (2016). Melatonin inhibits MMP-9 transactivation and renal cell carcinoma metastasis by suppressing Akt-MAPKs pathway and NF-κB DNA-binding activity. J Pineal Res.

